# Phenotype profiling of *Rhizobium leguminosarum* bv. *trifolii* clover nodule isolates reveal their both versatile and specialized metabolic capabilities

**DOI:** 10.1007/s00203-013-0874-x

**Published:** 2013-02-16

**Authors:** Andrzej Mazur, Grażyna Stasiak, Jerzy Wielbo, Piotr Koper, Agnieszka Kubik-Komar, Anna Skorupska

**Affiliations:** 1 Department of Genetics and Microbiology, Maria Curie-Skłodowska University, Akademicka 19, 20-033 Lublin, Poland; 2 Chair of Applied Mathematics and Informatics, Lublin University of Life Sciences, Akademicka 13, 20-950 Lublin, Poland

**Keywords:** *Rhizobium leguminosarum*, Biolog metabolic profiling, Symbiosis

## Abstract

*Rhizobium leguminosarum* bv. *trifolii* (*Rlt*) are soil bacteria inducing nodules on clover, where they fix nitrogen. Genome organization analyses of 22 *Rlt* clover nodule isolates showed that they contained 3–6 plasmids and majority of them possessed large (>1 Mb), chromid-like replicon with exception of four *Rlt* strains. The Biolog phenotypic profiling comprising utilization of C, N, P, and S sources and tolerance to osmolytes and pH revealed metabolic versatility of the *Rlt* strains. Statistical analyses of our results showed a clear bias toward specific metabolic preferences, tolerance to unfavorable osmotic conditions, and increased nodulation activity of the strains having smaller amount of extrachromosomal DNA. The K5.4 and K4.15 lacking a large megaplasmid possessed substantially diverse metabolism and belonged to effective clover inoculants. In conclusion, besides overall metabolic versatility, some metabolic specialization may enable rhizobia to persist in variable environments and to compete successfully with other bacteria.

**Electronic supplementary material:**

The online version of this article (doi:10.1007/s00203-013-0874-x) contains supplementary material, which is available to authorized users.

## Introduction

The rhizosphere is the microbe-rich zone surrounding plant roots. It is a dynamic environment, where resource distribution varies spatially and temporally, with plants providing a plethora of carbon and energy sources that significantly affect the populations of microorganisms in a manner specific to the host (Bais et al. 2006; Bertin et al. 2003; Haichar et al. 2008; Ramachandran et al. 2011). Bacteria must have evolved a wide variety of metabolic strategies to cope with such a dynamic environment. Moreover, they are often faced with unfavorable conditions such as osmotic stress, drought, heavy metals, and other toxins, as well as temperature changes. It is assumed that the structural and functional diversity of microbial communities in the rhizosphere is influenced by many biotic and abiotic factors (Berg and Smalla 2009).

Rhizobia are an example of bacteria, which can survive in the soil where resources are scarce and diverse, and compete for nutrients with other bacteria present in the host plant rhizosphere (El Yahyaoui et al. 2004; Prell and Poole 2006). They can also enter into a beneficial symbiosis with legumes in a highly specialized environment—the plant cell (Cai et al. 2009; Duodu et al. 2009; Faure et al. 2009). Rhizobia form nodules on the roots of their host legume plants. In exchange for carbohydrates provided by the plant, they fix atmospheric nitrogen and deliver reduced nitrogen compounds to their host (Gibson et al. 2008). Since rhizobia are found in different and complex environments, such as the soil, the rhizosphere, or plant cells, it is expected that they are capable of utilizing many different compounds.

Soil bacteria (such as rhizobia) have complex and large, >6 Mb, genomes that reflect their diverse metabolic capabilities (Konstantinidis and Tiedje 2004). Such genomes are presumably ecologically advantageous in challenging environments. Thus, genome size and content could largely result from environmental pressure and bacterial adaptation to soil conditions (Barnett and Fisher 2006; Bentley and Parkhill 2004; Konstantinidis and Tiedje 2004; MacLean et al. 2007). The sequenced rhizobial genomes usually consist of a single circular chromosome and a set of plasmids, whose size ranges from several kb to Mb (Barran et al. 2001; Galibert et al. 2001; González et al. 2006; Reeve et al. 2010a, b; Watson and Heys 2006; Young et al. 2006). Genomic content of rhizobia can be divided into two groups: the core genome, comprising genes present in all strains, and the accessory genome, consisting of unique or strain-specific genes (Young et al. 2006). The accessory genome comprises genes responsible for the symbiotic interaction with legume plants, which are typically located on one of the plasmids, called the symbiotic plasmid, or incorporated into the bacterial chromosome as symbiotic islands (Palacios and Newton 2005; Sullivan et al. 2002). Recently, in some rhizobia and other bacteria, extrachromosomal replicons called “chromids” were reported, with intermediate characteristics of the chromosome and plasmids (Harrison et al. 2010). Chromids are secondary replicons with plasmid maintenance and replication systems but bear some core genes and a far higher number of accessory genes than the chromosome. These genes are shared by chromids of other species in the same genus (Harrison et al. 2010).

*Rhizobium leguminosarum* bv. *trifolii* (*Rlt*) is a microsymbiont of clover and is able to fix atmospheric nitrogen in root nodules of this plant. Our previous studies of *Rlt* isolates from root nodules of clover plants growing at the same site showed a substantial divergence of their genome organization, especially as regards the plasmid DNA content (Mazur et al. 2011). The isolates harbored between 3 and 6 plasmids with sizes from ca. 150 to 1,380 kb. The total approximated amount of extrachromosomal DNA in the sampled *Rlt* strains ranged from 1,890 (e.g., K3.6) kb to 3,250 kb (e.g., K4.13). Furthermore, most of the strains had large (>1 Mb), chromid-like replicon with the exception of four *Rlt* strains K3.6, K3.16, K4.15, and K5.4, in which this type of replicon was substantially smaller (Mazur et al. 2011). Despite the high variability in the number and size of plasmids in the studied strains, conservation of the location as well as the dynamic distribution of the individual genes (especially replication genes) in a specific genome compartment were demonstrated. Sequence divergence of particular genes was linked with their location in a given genome compartment, that is, the chromosome, chromid-like replicons, and plasmids. We also showed that the plasmid genes were less adapted to the host genome than the chromosome and the chromid-like genes (Mazur et al. 2011). Currently, the knowledge of how this genomic diversity is correlated with phenotype differentiation and strains adaptation to the challenging environment is fragmentary; however, a number of high-throughput phenotype arrays are being used for functional characterization of genes of model bacteria (AbuOun et al. 2009; Rodrigues et al. 2011; Sabarly et al. 2011). In the previous studies of metabolic variability within the *Rlt* strains, we have demonstrated a prevalence of metabolically versatile strains, that is, not specializing in utilization of any group of carbon sources (Wielbo et al. 2010). Metabolic versatility as regards nutritional requirements was not directly advantageous for effectiveness in the symbiotic interactions with clover: metabolically specialized rhizobia were more effective in symbiosis but were rarely occurring in the population.

The purpose of this study was to extend and deepen the analyses of metabolic capacities of the previously genetically characterized 22 *Rlt* strains by their physiological profiling followed by statistical analyses. We employed Phenotype Biolog MicroArray (PM) technology, to test the utilization of numerous sources of carbon, nitrogen, phosphorus, and sulfur, as well as tolerance to osmolytes and different pH conditions in the clover nodule isolates, which was followed by plant tests to estimate their symbiotic activity. The potential interrelation between observed phenotypic traits and amount of extrachromosomal DNA in the sampled *Rlt* strains was assessed by comprehensive statistical analyses.

## Materials and methods

### *R. leguminosarum* bv. *trifolii* (*Rlt*) strains used in this study

*Rlt* isolates were obtained from nodules of red clover (*Trifolium pratense* L. cv. Dajana) growing in sandy loam (N:P:K 0.157:0.014:0.013 %), as described previously (Wielbo et al. 2010). Briefly, plants were grown on 1 m^2^ plot for six weeks between May and June 2008. Afterward, 10 randomly chosen clover plants growing in each other’s vicinity were harvested, the nodules were collected, surface-sterilized, crushed, and their content plated on 79CA medium (Vincent 1970). Pure cultures were used in further experiments. From the collection of 126 isolates, 22 *Rlt* strains previously characterized as differing in the plasmid pattern (Mazur et al. 2011) were submitted to Biolog phenotype profiling. *Rlt* strains were grown and maintained in 79CA or tryptone–yeast (TY) complex media at 28 °C.

### Biolog phenotypic assays

Assays of utilization of carbon (C), nitrogen (N), phosphorus (P), and sulfur (S) sources by *Rlt* isolates and tolerance to different osmotic and pH conditions were performed using Biolog GN2, PM2A, PM3B, PM4A, PM9, and PM10 microplates, Biolog Inc. (Hayward, CA). GN2 and PM2A plates were used to study C sources metabolism, PM3B plates to assess N metabolism, and PM4A for P and S sources, respectively. In addition, PM9 and PM10 plates were used to test the growth under various stress conditions and different pH (Bochner et al. 2008; Bochner 2009). The number of all the possible conditions assayed in the four types of microplates (GN2, PM2A, PM3B, and PM4A) was 379.

Bacteria growing overnight at 28 °C in TY medium were pelleted and washed twice with sterile water. After that, for GN2 plates, the pellet was diluted in water to an initial optical density at 550 nm (OD_550_) of 0.1, and a 100 μl suspension of rhizobia was inoculated into each well of the GN2 microplate. For the remaining plates (PM2A, PM3B, PM4A, PM9, and PM10), the washed bacteria were suspended in 10 ml of inoculating fluid (IF-0a) and OD_550_ was adjusted to 0.5. The bacterial suspensions were further diluted into 12 ml (per plate) of a relevant inoculating fluid. IF-0a inoculating fluid supplemented with Dilworth’s vitamins was used for PM2A plate; IF-0a with 20 mM sodium pyruvate and vitamins was used for plates PM3B and PM4A; IF-10 Base was used for PM9 and PM10 plates, respectively. The prepared bacterial suspensions were inoculated into wells of the appropriate microplates. The cells were incubated for 72 h at 28 °C, and color development (absorbance at 590 and 750 nm) in the wells was monitored using Benchmark Plus™ microplate reader (Bio-Rad Laboratories, USA). The conversion of colorless tetrazolium violet to a purple-colored compound meant normal process of respiration (positive phenotype), whereas when the phenotype was negative, the wells remained colorless. The optical density (OD) values of Biolog microplate wells were corrected using background color developed in the control well A1.

### Plant tests

Red clover seeds (*Trifolium pratense* L. cv. Dajana) were surface-sterilized and germinated on nitrogen-free Fåhraeus medium. Clover seedlings were inoculated with 0.2 ml of *Rlt* cell suspension at an approximate density of 1.0 × 10^9^ cells/ml and were grown (one per tube) in a greenhouse under natural light supplemented with artificial light (14/10-h light/dark) regime and at 24/19 °C day/night temperature. After 4 weeks, the plants were harvested, the nodules were counted, and fresh masses of shoots and roots were estimated. For each strain, 20 clover plants were used.

### Statistical data analysis

For principal component analyses (PCAs), the results of Biolog test were coded in the binary system, and PCA with varimax rotation (Morrison 1990) was used to analyze bacterial capability of utilizing particular substrates or group of substrates. This method allowed us to transform the numerous variables (utilization of 19 groups of substrates), possibly correlated as well, into a small group of uncorrelated factors (PCs). Factor loadings in PCAs (Table S3) were the correlation coefficients between the original variables (metabolic substrates) and the obtained factors called PC1, PC2, etc.

The relationships between (a) the organization of *Rlt* genome and the number of utilized substrates classified into different PCs, as well as (b) the organization of *Rlt* genome and the symbiotic properties of strains, were studied using one-way analysis of variance (ANOVA), which was performed using Statistica software.

## Results

### Phenotypic profiling of *Rlt* strains

The *Rlt* strains involved in this study have been previously analyzed with respect to genome organization and genetic diversity (Mazur et al. 2011). Currently, 22 *Rlt* strains were tested for metabolic potential with various compounds used as the sole sources of carbon (C), nitrogen (N), sulfur (S), and phosphorus (P) (Table S1). To gain general overview of metabolic divergence of *Rlt* strains and to simplify initial examination, the substrates contained in GN2, PM2A, PM3B, PM4A microplates were arbitrarily divided into 19 groups: monosaccharides, oligosaccharides, polysaccharides, sugar alcohols, modified sugars, phospho-sugars, d-amino acids, l-amino acids, modified amino acids, oligopeptides (dipeptides mainly), amines, glycosides, carboxylic acids, sugar acids, modified carboxylic acids, nitrogen bases, nucleosides and nucleotides, inorganic compounds and miscellaneous, that is, other organic compounds not classified into the aforementioned groups (herein referred to as “other organic”) (Table S2). The number of the various substrates taken into account in this analysis was 301 and was less than the number of all the possible compounds assayed in the four types of microplates (379 compounds) and resulted from exclusion of some repeated substrates on different plates.

**Fig. 1:**
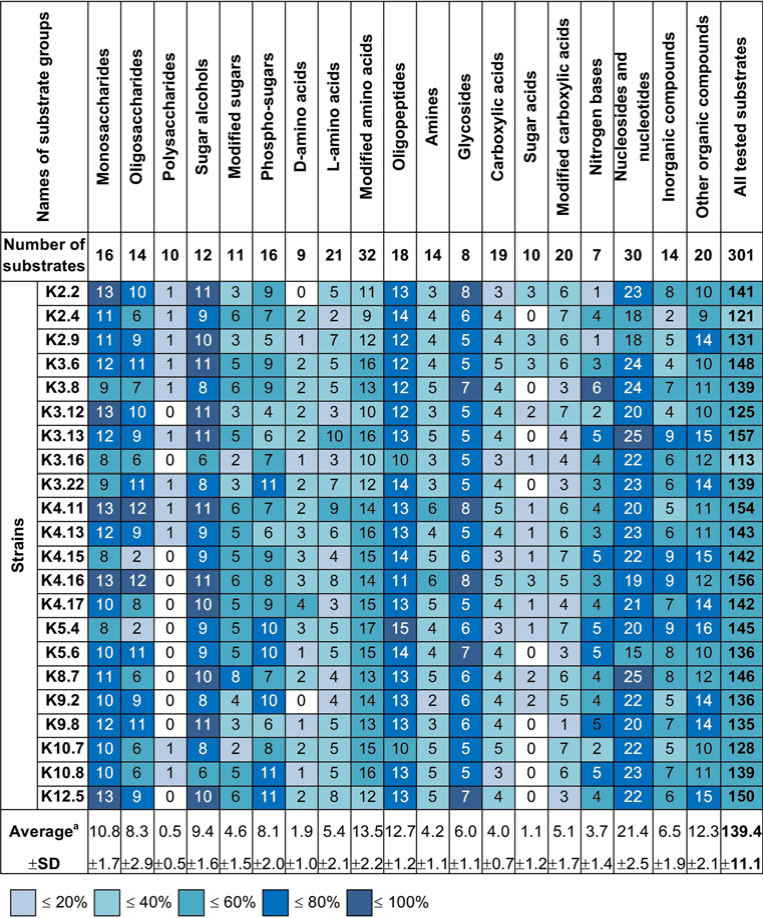
Phenotypic profiles of *Rlt* strains on Biolog GN2, PM2a, PM3b, and PM4a microplates. The substrates were arbitrarily divided into 19 groups. The number of substrates present in each group and average substrates utilization was shown. The *colors* indicate the percentage of utilized substrates of individual groups. ±SD—standard deviation. ^a^Average number of substrates of each group utilized by individual strains

Positive phenotypes for at least one strain were observed for 206 sources (68.4 %). Of all the compounds, 71 (23.6 %) were metabolized by all the tested *Rlt* strains. The individual *Rlt* isolates metabolized from 113 (37.5 %) to 157 (52.2 %) substrates (Fig. 1). The polysaccharides were metabolized at the lowest extent; merely half of the strains used 1 of 10 polysaccharide substrates. Similarly, sugar acids were rarely utilized (up to 3 of 10 compounds) and nine strains used none of them. d-amino acids were also rarely used (up to 4 of 9 compounds) and two strains used none of them (Fig. 1). On the other hand, the most commonly utilized substrates were sugar alcohols, glycosides, nucleosides and nucleotides, and oligopeptides (comprising mainly dipeptides), with an average utilization rate of 78.0, 74.4, 71.4, and 70.7 %, respectively (Fig. 1). Monosaccharides were quite well utilized by the *Rlt* isolates; from among 16 such substrates, the tested strains used 8–13 of them, with an average utilization at 67.6 %. The most metabolically versatile strains (K3.13, K4.16, K4.11, K12.5) used approximately half of the tested substrates (150–157). The lowest number of substrates (113) was utilized by K3.16 strain (Fig. 1).

Strain tolerance to osmotic stress was analyzed with PM9 microplates (Table 1). Tolerance levels to different concentrations of NaCl, sodium sulfate, ethylene glycol, sodium formate, urea, sodium lactate, sodium phosphate, ammonium sulfate, sodium nitrate, and sodium nitrite were examined. In general, the *Rlt* strains were extremely sensitive to NaCl and Na_2_SO_4_, with the exception of K4.15, K5.4, and K9.8 strains, which tolerated the presence of 1 % NaCl and 2 % Na_2_SO_4_, respectively. Majority of the strains showed no metabolic activity in the presence of sodium formate and sodium lactate (Table 1). Up to 2 % urea did not disturb the respiration of eight strains, and K4.15 and K5.4 were able to tolerate as much as 4 % urea (Table 1). All the strains displayed high tolerance to ethylene glycol, sodium phosphate, ammonium sulfate, and sodium nitrate; several strains could tolerate these chemicals even at the highest tested concentrations. Sodium nitrite was generally well tolerated in low concentration (10–40 mM). K4.15 and even more so K5.4 were distinct, with their higher tolerance to several of the tested compounds (Table 1).

**Table 1:** The *Rlt* strains responses to osmotic stress and pH in Biolog PM9 and PM10 microplates

Osmotic stress factor or pH value	Sodium chloride	Sodium sulfate	Ethylene glycol	Sodium formate	Urea	Sodium lactate	Sodium phosphate	Ammonium sulfate	Sodium nitrate	Sodium nitrite	pH
Tested range	1–10 %	2–5 %	5–20 %	1–6 %	2–7 %	1–12 %	20–200 mM	10–100 mM	10–100 mM	10–100 mM	3.5–10.0
Strains											
K2.2	–	–	15	–	–	–	100	50	60	10	5.0–7.0
K2.4	–	–	5	–	–	–	50	20	60	10	5.5–6.0
K2.9	–	–	15	–	–	–	100	50	60	10	5.5–8.0
K3.6	–	–	20	–	–	–	50	50	100	10	5.5–7.0
K3.8	–	–	15	–	–	–	100	50	100	20	5.5–7.0
K3.12	–	–	20	–	2	1	100	100	60	10	5.0–7.0
K3.13	–	–	20	–	–	–	100	50	100	20	5.5–7.0
K3.16	–	–	5	–	2	–	100	50	40	–	5.5–8.5
K3.22	–	–	20	–	3	–	100	50	60	10	5.5–7.0
K4.11	–	–	10	1	2	–	100	100	40	20	5.0–6.0
K4.13	–	–	15	–	–	–	50	50	60	10	5.5–7.0
K4.15	1	2	15	–	4	1	100	50	100	20	5.5–6.0
K4.16	–	–	20	–	–	–	100	50	40	–	5.0–7.0
K4.17	–	–	15	–	–	1	100	50	80	20	5.5–7.0
K5.4	1	4	20	–	4	1	200	100	100	40	5.0–8.5
K5.6	–	–	20	–	2	–	200	100	100	20	5.5–7.0
K8.7	–	–	20	–	–	–	50	50	40	10	5.5–6.0
K9.2	–	–	15	–	–	1	50	20	40	–	5.5–6.0
K9.8	1	2	20	–	2	–	50	50	40	10	5.5–8.0
K10.7	–	–	15	–	–	1	50	20	60	–	5.5–6.0
K10.8	–	–	20	1	–	–	100	50	100	10	5.5–7.0
K12.5	–	–	20	–	–	–	50	50	60	10	5.5–7.0

The numbers indicate the maximum concentration of a given compound or pH value range, in which positive phenotype on Biolog microplates was observed

Finally, metabolic activity of the *Rlt* isolates over a broad range of pH 3.5–10 was determined using PM10 microplates. The strains were viable within pH range from 5.0 to 8.5; however, some of them showed very narrow tolerance to pH changes, that is, within 0.5 units (pH 5.5–6.0). Majority of *Rlt* strains tolerated pH from slightly acidic (pH 5.0–5.5) to neutral (pH 7), only few of them showed metabolic activity also at slightly alkaline pH (pH 8.0–8.5). Strain K5.4 was the most resistant to pH changes and was metabolically active at pH 5.0–8.5 (Table 1).

**Fig. 2:**
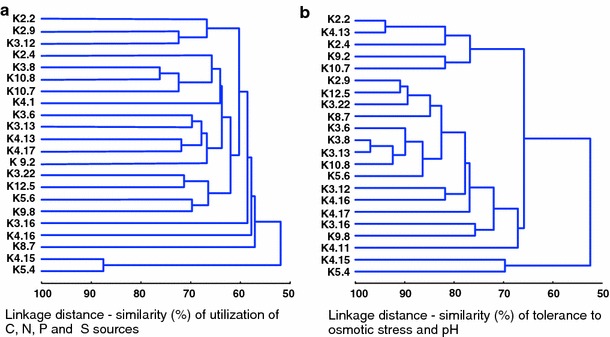
Cluster analysis-based dendrograms (UPGMA method) showing phenotypic diversity of the sampled *Rlt* strains with respect to utilization nutrition compounds (C, N, P, S) (**a**), and tolerance to osmotic stresses and pH (**b**)

The results of metabolic capability assays of the individual *Rlt* strains were subjected to UPGMA clustering analysis (Fig. 2a). First, the number of various nutrition sources (C, N, P, and S) utilized by the individual strains was used for grouping, taking into account only the discriminatory variables. Most of the strains (17/22) formed a large group of metabolically similar isolates. Three strains (K3.16, K4.16, and K8.7) clustered outside of this group, revealing slightly distinct metabolism, while two other, that is, K4.15 and K5.4, split up the remaining strains by forming a separate branch in the dendrogram (Fig. 2a). The metabolic profiles of K4.15 and K5.4 were highly similar to each other but significantly differed from the remaining strains.

In the dendrogram based on Biolog results of tolerance to osmotic stresses and pH sensitivity, most of the *Rlt* isolates also formed one large group, further subdivided into two clusters of strains (Fig. 2b). Once again, K4.15 and K5.4, most tolerant to osmotic stress and variations in pH, clustered in a separate branch of the dendrogram (Fig. 2b).

Overall, majority of the sampled strains revealed some degree of versatility and, simultaneously, significant similarity in metabolic profiles with respect to the number of utilized nutrition sources and tolerance to stresses and pH, except for a few strains evidently distinct from the rest.

### Principal component analysis (PCA)

To qualitatively analyze metabolic differentiation between the sampled *Rlt* strains, the results of the Biolog assay were subjected to PCA. Two types of analyzes were performed. Firstly, 19 previously arbitrary distinguished groups of energy sources (comprising 301 compounds) were classified into principal components (PCs). This PCA will be referred as general (PCA^G^) throughout the manuscript. The main criterion for the classification of every substrate group to the individual principal component was high correlation coefficient (positive or negative) of the substrate group with a given principal component and low rates for the remaining principal components. PC1^G^ to PC7^G^ were responsible for 81.12 % of the total variance of metabolic capabilities of *Rlt* strains (Table S3A). The PC1^G^ included monosaccharides, oligosaccharides, sugar alcohols, and carboxylic acids; PC2^G^ constituents were modified sugars, d-amino acids, and amines; inorganic compounds and “other organic” were classified into PC3^G^; PC4^G^ comprised sugar acids, modified carboxylic acids, and nitrogen bases; PC5^G^—nucleosides and nucleotides; PC6^G^—glycosides; PC7^G^ contained oligopeptides (Table S3A).

**Fig. 3:**
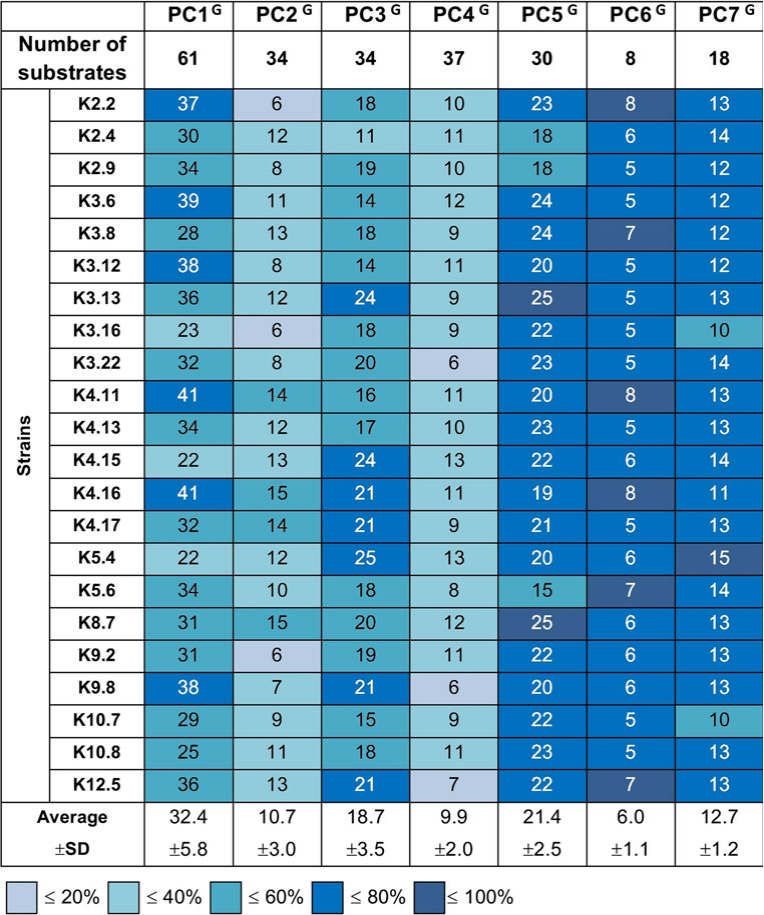
Number of substrates utilized by individual *Rlt* strains, grouped in seven most important principal components (PC) in PCA^G^. The *colors* indicate the percentage of utilized substrates of individual PCs

Only the first four principal components (PC1^G^–PC4^G^) were composed of more than one group of substrates, in total encompassing twelve groups of compounds, and contributed to 60.37 % of variance of the tested strains. PC1^G^ containing 61 compounds (Fig. 3) was responsible for 22.02 % of the metabolic variance of *Rlt* isolates (Table S3A). *Rlt* strains metabolized from 22 to 41 substrates classified into PC1^G^. K4.11 and K4.16 strains utilized the most compounds included into the PC1^G^, while the K3.16, K4.15, and K5.4 used few of these substrates (Fig. 3).

PC2^G^ included 34 compounds classified into three groups (modified sugars, d-amino acids, and amines) and contributed to 17.97 % of variance (Table S3A). The *Rlt* isolates metabolized from 6 to 15 substrates from PC2^G^ (on average 31.4 % of substrates) (Fig. 3). Variance in metabolic capabilities of another 11.07 % of *Rlt* strains corresponded to PC3^G^, which included inorganic compounds and “other organic compounds,” together constituting a group of 34 substrates. Average utilization of these compounds was equal to 55.1 %, reaching 73.5 % for K5.4, K4.15, and K3.13 strains, respectively. PC4^G^ was responsible for 9.31 % of variance and comprised 37 compounds: 10 sugar acids, 20 modified carboxylic acids, and 7 nitrogen bases. Compounds classified to PC4^G^ were relatively seldom metabolized by the strains, on average 16.2 %.

The PC5^G^, PC6^G^, and PC7^G^ encompassed only single of the previously described groups of compounds, that is, nucleosides and nucleotides, glycosides, and oligopeptides (dipepetides mainly), respectively (Table S3A), and these principal components did not contribute substantially to the metabolic diversity of the tested strains.

In the performed PCA^G^, the individual PCs encompassed groups of substrates, which may be both sources of carbon, nitrogen, sulfur, and phosphorus. To find out whether the specific metabolism of carbon, nitrogen, sulfur, or phosphorus is responsible for the *Rlt* strains metabolic variance, another PCA was done in which utilization of (a) carbon sources, (b) nitrogen sources, and (c) phosphorus and sulfur sources were studied separately (PCA^C^, PCA^N^, PCA^P/S^). Classification of individual substrates remained almost the same as in previous PCA^G^, with minor exceptions. Substrates previously included in the group of nucleosides and nucleotides were now classified as nucleosides group (when carbon or nitrogen sources were studied) or as nucleotides group (in phosphorus and sulfur sources utilization analysis). Moreover, in PCA^P/S^ thio-*β*-d-glucose, previously belonging to the group of modified sugars was included into other organic compounds. In the PCA concerning utilization of carbon, nitrogen, phosphorous, and sulfur sources, 15, 11, and 8 groups of substrates were analyzed, respectively. Furthermore, this kind of analysis allowed us to consider utilization of all possible 379 compounds without excluding of any repeating sources.

In PCA focusing on carbon sources—PCA^C^ (comprising 15 groups of substrates)—six principal components were identified and the most important PC1^C^, PC2^C^, and PC3^C^ together contributed to 59.5 % of variance of tested strains (Table S3B). PC1^C^ was mainly composed of monosaccharides, oligosaccharides, carboxylic acids, nucleosides, as well as sugar alcohols (with lower correlation coefficient). PC2^C^ contained l-amino acids and oligopeptides, whereas PC3^C^ comprised sugar acids and modified carboxylic acids.

**Fig. 4:**
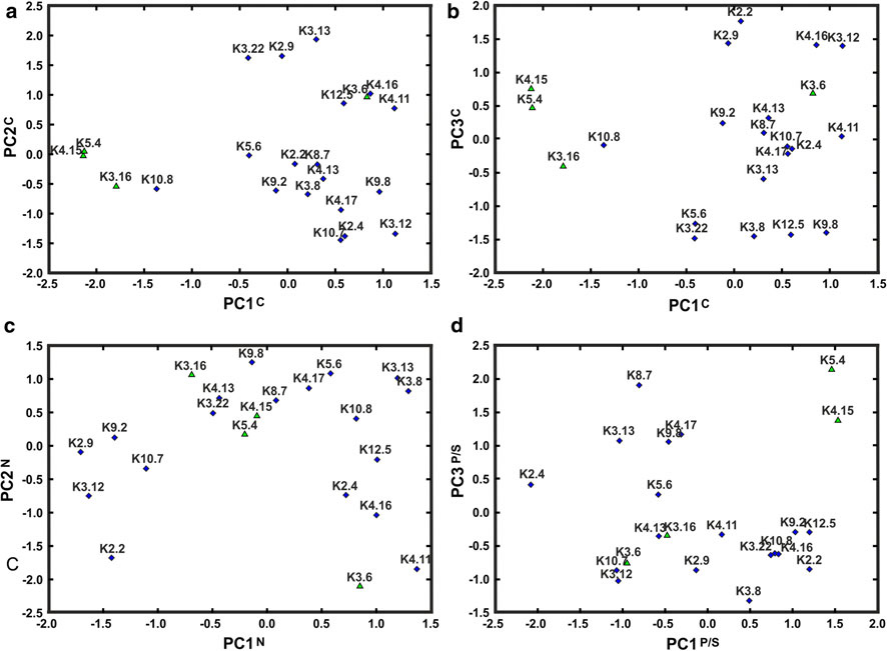
The scatterplots of selected 22 *Rlt* strains in **a** the principal component 1 (PC1^C^) and principal component 2 (PC2^C^), **b** the principal component 1 (PC1^C^) and principal component 3 (PC3^C^), **c** the principal component 1 (PC1 ^N^) and principal component 2 (PC2 ^N^), **d** the principal component 1 (PC1^P/S^) and principal component 3 (PC3^P/S^) coordinate system. **a** Numbers located close to the *symbols* are individual strain designation. The strains lacking large (>1 Mb) replicon are depicted by *triangles*

The scatterplots of *Rlt* strains in the PC1^C^ and PC2^C^, as well as in PC1^C^ and PC3^C^ coordinate system (Fig. 4a, b) showed that some strains (K4.15, K5.4, K3.16, K10.8) differed substantially in the utilization of substrates belonging to PC1^C^ group, whereas their abilities to utilize compounds classified into PC2^C^ or PC3^C^ were in the range of other strains (Fig. 4a, b).

When nitrogen sources (11 groups of substrates) were submitted for PCA (PCA^N^), only five principal components were identified, with the most important PC1 ^N^, PC2 ^N^, and PC3 ^N^, which contributed together to 66.8 % of metabolic variance of studied strains (Table S3C). PC1 ^N^ was mainly composed of modified sugars, l-amino acids, amines, and nitrogen bases (with lower correlation coefficient). PC2 ^N^ contained nucleosides, other organic compounds, and inorganic nitrogen sources (with lower correlation coefficient). PC3 ^N^ was highly correlated only with oligopeptides (dipepetides mainly) (Table S3C). Strains, which differed substantially from others in carbon metabolism revealed comparable nitrogen metabolic properties to other strains from studied group (Fig. 4c).

Finally, phosphorus and sulfur sources utilization was studied by the PCA (PCA^P/S^), in which four principal components were identified (Table S3D). PC1^P/S^ was mainly composed of inorganic P and S compounds as well as phospho-sugars and other organic compounds (with lower correlation coefficients) and contributed to 33.7 % of variance. PC2^P/S^ contained l-amino acids and modified amino acids, resulting in further 21.8 % of metabolic variance (Table S3D). PC3^P/S^ comprised mainly d-amino acids and oligopeptides, PC4^P/S^ comprised only nucleotides contributing, respectively, to 12.6 % and 11.9 % of metabolic variance of studied strains. It was observed that some of the strains, namely K4.15 and K5.4, differed mostly in the utilization of compounds classified to PC1^P/S^ and PC3^P/S^ (Fig. 4d). These strains diverged substantially also in carbon metabolism analyzed in PCA^C^ (Fig. 4a, b).

### Correlation between genome complexity and metabolic activity of *Rlt* strains

According to the data obtained with Biolog microplates, majority of the *Rlt* strains had similar metabolic profiles, as well as tolerance to osmotic stresses and pH, except for a few strains, which were remarkably distinct. K3.16, K4.15, K4.16, K5.4, and K8.7 did not cluster together with the remaining strains, forming separate branches in UPGMA dendrogram based on the number of utilized compounds (Fig. 2a). Furthermore, K4.15 and K5.4 split the UPGMA dendrogram based on tolerance to osmotic stresses and pH. K3.16, K4.15, and K5.4 utilized the lowest number of compounds belonging to PC1^G^. On the other hand, substrates well metabolized by K4.15 and K5.4 belonged to PC4^G^ and PC3^G^ (inorganic compounds and “other organic compounds”) (Fig. 3), indicating metabolic dissimilarity of these strains. Strains K4.15, K5.4, K3.16, K10.8 showed differences in the utilization of monosaccharides, oligosaccharides, carboxylic acids, nucleosides, sugar alcohols, and modified sugars as carbon sources (PC1^C^). Furthermore, in the cases of K4.15 and K5.4 differences in the utilization of inorganic compounds, phospho-sugars, other organic compounds, d-amino acids, and oligopeptides as phosphorus and sulfur sources (PC1^P/S^, PC3^P/S^) were observed. Interestingly, the metabolically distinct K3.16, K4.15, and K5.4 represent the subgroup of the *Rlt* strains lacking a very large plasmid (>1 Mb).

We subsequently focused on the potential relationship between the genome complexity and metabolic capabilities of the sampled *Rlt* strains. Significant difference (*p* < 0.05) was observed in the number of utilized substrates classified into general PC1^G^ and PC4^G^ for *Rlt* isolates, which possessed a very large plasmid (>1 Mb) versus those which did not have such replicon. In general, the strains lacking the very large replicons utilized significantly lower number of PC1^G^ classified compounds, especially monosaccharides and oligosaccharides, in comparison with strains having such plasmids (an average 26.5 versus 33.7). Simultaneously, the same strains demonstrated higher metabolic versatility in the utilization of various sugar acids, modified carboxylic acids, and nitrogen bases of PC4^G^-classified compounds (an average 11.8 versus 9.5). In the subgroup of *Rlt* strains without the very large plasmids, K4.15 and K5.4 were of special interest. These strains not only metabolized less substrates of PC1^G^ in comparison with other strains but, concomitantly, utilized more inorganic compounds and other organic compounds of PC3^G^ (an average 24.5 versus 18.2), as well as sugar acids, modified carboxylic acids, and nitrogen bases of PC4^G^ (13 versus 9.6). In the PCAs taking into account utilization C, N, P, and S sources, significant difference (*p* < 0.05) was also observed in the number of utilized carbon sources classified into PC1^C^ for *Rlt* isolates differing in the possession of >1 Mb plasmid. The strains lacking the very large replicons utilized significantly lower number of PC1^C^ classified compounds, that is, monosaccharides, oligosaccharides, carboxylic acids, nucleosides, as well as sugar alcohols and modified sugars in comparison with strains having such plasmids (an average 22.3 vs. 34.9). Concomitantly, the strains lacking >1 Mb plasmid utilized more inorganic compounds, phospho-sugars, other organic compounds, d-amino acids, and oligopeptides included into PC1^P/S^ and PC3^P/S^ (an average 28.3 vs. 21.9, *p* < 0.1).

K4.15 and K5.4 strains were also less sensitive to unfavorable osmotic conditions in comparison with the others; K5.4 was found to be the most tolerant strain within the sampled group. However, we did not find any significant correlation between the tolerance to osmotic stresses, or a wide range of pH, and the size of the extrachromosomal genome or the presence of the very large plasmids.

### Symbiotic characteristics of *Rlt* strains

**Fig. 5:**
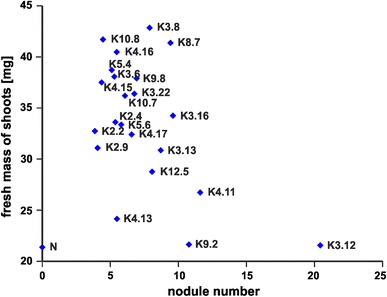
Fresh mass and roots nodule number of clover inoculated with the sampled *Rlt* strains

Symbiotic efficiency of *Rlt* strains was examined in a laboratory plant test, in which red clover plants were inoculated with the relevant bacteria. Average shoot fresh mass ranged from 22 mg (K3.12 and K9.2) to more than 40 mg/plant (in clovers infected with K3.8, K4.16, K8.7, and K10.8 strains) (Fig. 5). Clover plants inoculated with *Rlt* displayed large discrepancy in the number of root nodules, from 4 nodules (K2.9, K2.2, K4.15) to 10–20 nodules/plant (for clovers infected with K4.11, K3.12, and K9.2 strains). An increased nodule number was not correlated with an increase in shoots mass of plants. Interestingly, significant differences were noted for the wet masses of clover shoots. It was observed that plants inoculated with strains without the very large plasmids had a significantly higher fresh shoot mass than plants infected with strains harboring such plasmid (an average 37.5 and 32.8 mg/plant, *p* < 0.05, respectively). Thus, the most metabolically distinct K5.4 and K4.15 strains belonged to the group of symbiotically effective inoculants (Fig. 5).

## Discussion

The metabolic properties of an organism may contribute toward a particular niche adaptation. Metabolic differences that have enabled rhizobia to adapt to specific environments, such as the soil and plant tissues, are poorly understood but may be key in understanding how bacteria survive and compete for host’s nodulation. The *Rlt* strains included in this study could be considered champions in clover nodulation competition, thus might provide valuable information about bacteria metabolic approaches helpful for root colonization. The strains have been previously characterized on a genetic level and the diversity of their genomic organization had been described (Mazur et al. 2011). From among 22 *Rlt* strains, in four of them, the largest chromid-like replicon was substantially smaller than 1 Mb and the total amount of extrachromosomal DNA (an average 2,359 kb) was lower by about 551 kb than in the remaining strains (an average 2,910 kb). The question was whether these differences in amount of extrachromosomal DNA and overall genomic heterogeneity of the strains reflect the metabolic potential and symbiotic activity of the sampled rhizobia. Therefore, we compared substrate utilization data gathered from high-throughput phenotyping microarrays for a set of environmental isolates, which differed in genomic architecture and plasmid patterns.

Metabolic profiling of 22 *Rlt* isolates was performed using Biolog microplates, comprising analyses of the utilization of nutritional compounds (C, N, P, S), as well as tolerance to osmolytes and different pH conditions. The obtained patterns of phenotypes suggested that *Rlt* strains are capable of utilizing a variety of metabolic substrates (68.4 % of the tested sources), with significant preferences for sugar alcohols, glycosides, nucleosides, nucleotides, and oligopeptides (comprising mainly dipeptides), with an average utilization of ~73 % of the compounds. Monosaccharides were also quite well utilized with an average of 67.6 % and specific sugars, such as d-fructose, l-fucose, d-glucose, d-psicose, and l-, d-arabinose, were utilized by all 22 strains. All the tested strains also utilized sucrose and d-trehalose, and up to 50 % of oligopeptides (9 of 18) or glycosides (4 of 8), respectively. About 30 % of the tested nucleotides and nucleosides were metabolized by all strains. In general, the phenotypic profiling in conjunction with statistical analyses revealed versatility of the sampled *Rlt* strains. Only a few strains were metabolically distinct in relation to others, with two, namely K5.4 and K4.15, remarkably dissimilar. In comparison with the remaining isolates, these strains metabolized less monosaccharides and oligosaccharides, and more inorganic and other organic compounds, as well as sugar acids, modified carboxylic acids, and nitrogen bases, respectively. With respect to the specific metabolism, K5.4 and K4.15 utilized less monosaccharides, oligosaccharides, carboxylic acids, nucleosides, as well as sugar alcohols and modified sugars as carbon sources and more inorganic compounds, phospho-sugars, other organic compounds, d-amino acids and oligopeptides (dipeptides) as P and S sources, but they did not differ substantially in nitrogen sources utilization.

Recent comparative transcriptomics studies of *R. leguminosarum* adaptation to rhizospheres of legumes (Ramachandran et al. 2011) provided valuable insight into potential metabolic strategies employed by bacteria for the colonization of a specific host plant. In general, the metabolism of *R. leguminosarum* in the rhizosphere was biased toward organic acids. Moreover, one of the strongest general metabolic responses was the induction of genes encoding proteins involved in the catabolism of phenylalanine and tyrosine, as well as transporter genes involved in the uptake of other aromatic compounds (Ramachandran et al. 2011). This could be simply explained by a possible abundance of these compounds in the natural environment of rhizobia—soil constituents, plant metabolites, or plant breakdown products can all be used as a source of carbon by the rhizosphere bacteria. Indeed, it has been demonstrated that soils are rich in organic acids (Bertin et al. 2003; Gaworzewska and Carlile 1982; Lugtenberg et al. 2001; Prell and Poole 2006).

Previously, it was postulated that the more metabolically versatile strains were more successful competitors in host plant nodulation (Wielbo et al. 2007). On the other hand, it was demonstrated that metabolic versatility with regard to nutritional requirements was not directly advantageous for effectiveness in the symbiotic interaction with clover: rhizobia with specialized metabolism were more effective in symbiosis but rarely occurred in the population (Wielbo et al. 2010). Interestingly, in our studies, the most metabolically diverse K5.4 and K4.15 strains, which were more specialized in the utilization of specified compounds, belonged to the “effective” group of clover inoculants. It was suggested that the utilization of nutrition compounds present in the soil may affect the ability of *R. leguminosarum* to compete in the pea rhizosphere (Ramachandran et al. 2011). Furthermore, Cai et al. (2009) have shown that host legumes may exude specific antimetabolites to fine-tune the bacterial population and enhance successful symbiosis with rhizobia. The plant host habitat, as well as root exudates, can shape the soil bacterial community structure by the generation of carbon sources available for microbial growth (Berg and Smalla 2009; Haichar et al. 2008), and bacteria must have evolved metabolic adaptations specific to individual plant species. Comparative genomic analyses of 14 sequenced rhizobial genomes revealed a large number of nitrogen, methane, sulfur, amino acid, vitamins, and cofactors metabolism orthologs. The diverse suite of metabolism pathways may be indicative of the ability to live in the complex rhizosphere environments, as well as to adapt to the nodule environment (Black et al. 2012).

Statistical analyses of the Biolog profiles of our strain collection showed a clear bias toward different phenotypic traits, such as metabolic preferences, sensitivity to unfavorable osmotic conditions, and nodulation activity of strains having smaller amount of extrachromosomal DNA, and as demonstrated previously, displaying a slightly different gene distribution (Mazur et al. 2011). However, the examples of K4.15 and K5.4 show that smaller amount of extrachromosomal DNA or differences in plasmids genes distribution do not have to be associated with weaker metabolic or adaptive potential. These metabolically distinct strains revealed also higher tolerance to osmotic stresses and a wide range of pH showing the specific metabolic adaptation to different environmental conditions. Plausibly, this level of adaptation is based on the regulation of several ATP-dependent transport systems acting pleiotropically, which are responsible for both potassium homeostasis or oligopeptide transport and whose action results in tolerance to osmolites (Prell et al. 2012; Sleator and Hill 2001). Concomitantly, there was no correlation between the tolerance to osmotic stress and the presence of a very large plasmid suggesting chromosomal or other plasmids location of the genes encoding these metabolic traits. In our previous study concerning genome organization of clover nodule isolates based on marker distribution (Mazur et al. 2011), K5.4 and K4.15 formed clearly separated group of monophyletic origin. It might suggest different evolutionary history of these strains explaining substantial differences in their metabolism. The strains were metabolically diverse but, nonetheless, belonged to effective clover inoculants. Thus, the relationships between genetic potential and metabolic capabilities are far more complex, especially in large, multipartite genomes.

A traditional concept in bacterial genetics states that housekeeping genes indispensable for basic metabolic function of the cell are usually assumed as chromosomally encoded, whereas those required for dealing with a challenging environment are located extrachromosomally, that is, on plasmids. Nevertheless, there are many examples of plasmid-encoded catabolic genes, which may contribute to the adaptiveness and competitiveness of this group of bacteria in the rhizosphere (Baldani et al. 1992; Brom et al. 2000; Ding et al. 2012; Oresnik et al. 1998; Yost et al. 2006). The post-genomic era has further enriched this view by discovery in bacteria secondary chromosomes or plasmids with a chromosome role, named chromids (Harrison et al. 2010; Landeta et al. 2011; MacLellan et al. 2004). Exceptions concerning possession of a gene (or gene sets), which contributes to cell viability, are especially noticeable in bacteria with multipartite genomes. A large segment of pSym replicon of *Sinorhizobium meliloti* is required for dulcitol, melibiose, raffinose, P-hydroxybutyrate, acetoacetate, protocatechuate, and quinate utilization (Charles and Finan 1990). Nearly 11 % of the genes in p42e of *R. etli* (González et al. 2006) are involved in primary metabolism, both in biosynthetic functions (cobalamin, cardiolipin, NAD, thiamine) and degradation (asparagine and melibiose) (Landeta et al. 2011). *panC* and *panB* genes located on p42f of *R. etli* are indispensable for pantothenate synthesis and growth in minimal medium (Villaseñor et al. 2011). Furthermore, the plasmid pRL8 of *R. leguminosarum* bv. *viciae* can be considered pea rhizosphere specific, enabling the adaptation of bacteria to its host in terms of adjusting of the microsymbiont metabolism to the rhizosphere resources of a particular plant (Ramachandran et al. 2011). The genes located on chromids are potentially as stable as those on the chromosomes and such replicons are usually difficult to eliminate from the cells (Landeta et al. 2011). Thus, chromids already carrying several essential genes may facilitate broadening of bacterial metabolic potential through acquisition of adaptive foreign genes by horizontal transfer and stabilizing the new set of advantageous genes in the genome. However, it seems obvious that for bacteria possessing multipartite genomes and persisting in various environments, the cooperation among all the replicons might be necessary for providing basic cellular function and competitiveness with other microorganisms.

## Electronic supplementary material

Below is the link to the electronic supplementary material.

Supplementary material 1 (XLSX 52 kb)

Supplementary material 2 (DOC 53 kb)

Supplementary material 3 (DOCX 25 kb)
