# Supplementary material for: Phenotype profiling of Rhizobium leguminosarum bv. trifolii clover nodule isolates reveal their both versatile and specialized metabolic capabilities
Source: Arch Microbiol. 2013 Feb 16;195(4):255–67. doi: 10.1007/s00203-013-0874-x (PMC3597991; doi:10.1007/s00203-013-0874-x)
Supplement: Supplementary file 2 — Supplementary material 2 (DOC 53 kb) [file 203_2013_874_MOESM2_ESM.doc]

**Supplementary Table S2** The substrates present in Biolog GN2, PM2A, PM3B, PM4A arbitrarily divided into 19 groups

| **Name of substrate group** | **Number of compounds classified in an individual groups** | **Compounds** |
| --- | --- | --- |
| Monosaccharides | 16 | l-arabinose, d-fructose, l-fucose, d-galactose, α-d-glucose, d-mannose, d-psicose, l-rhamnose, β-d-allose, d-arabinose, 2-deoxy-d-ribose, d-fucose, l-glucose, sedoheptulosan, l-sorbose, d-tagatose |
| Oligosaccharides | 14 | d-cellobiose, gentiobiose, α-d-lactose, lactulose, maltose, d-melibiose, sucrose, d-trehalose, turanose, 3-d-β-d-galactopyranosyl-d-arabinose, palatinose, d-raffinose, d-melezitose, stachyose |
| Polysaccharides | 10 | α-cyclodextrin, β-cyklodextrin, γ-cyklodextrin, dextrin, glycogen, chondroitin sulfate C, inulin, laminarin, mannan, pectin |
| Sugar alcohols | 12 | adonitol, d-arabitol, l-arabitol, i-erythritol, m-inositol, d-mannitol, d-sorbitol, xylitol, 2,3-butanediol, glycerol, lactitol, maltitol |
| Modified sugars | 11 | n-acetyl-d-galactosamine, n-acetyl-d-glucosamine, 3-methyl-glucose, n-acetyl-d-glucosaminitol, d-glucosamine, d-galactosamine, d-mannosamine, n-acetyl-d-mannosamine, 1-thio-β-d-glucose, glucuronamide, d,l-lactamide |
| Phospho-sugars | 16 | d,l-α-glycerol phosphate, d-glucose-1-phosphate, d-glucose-6-phosphate, 2-deoxy-d-glucose-6-phosphate, d-glucosamine-6-phosphate, 6-phospho-gluconic acid, d-mannose-1-phosphate, d-mannose-6-phosphate, trietyl phosphate, glycerol phosphate, carbamyl phosphate, d-2-phospho-glyceric acid, d-3-phospho-glyceric acid, inositol hexaphosphate, phospho-glycolic acid, phosphoenolpyruvate |
| D-amino acids | 9 | d-alanine, d-serine, d-asparagine, d-aspartic acid, d-glutamic acid, d-lysine, d-valine, d-cysteine, d-methionine |
| L-amino acids | 21 | l-alanine, l-asparagine, l-aspartic acid, l-glutamic acid, l-histidine, l-leucine, l-phenylalanine, l-proline, l-serine, l-threonine, l-arginine, glycine, l-isoleucine, l-lysine, l-methionine, l-valine, l-cysteine, l-glutamine, l-tryptophan, l-tyrosine, l-valine |
| Modified amino acids and amino acids derivatives | 32 | hydroxy-l-proline, l-ornithine, l-pyroglutamic acid, d,l-carnitine, γ-amino butyric acid, urocanic acid, l-homoserine, l-citrulline, n-acetyl-d,l-glutamic acid, n-phthaloyl-l-glutamic acid, cysteamine-s-phosphate, phospho-l-arginine, o-phospho-d-serine, o-phospho-l-serine, o-phospho-l-threonine, o-phospho-d-tyrosine, o-phospho-l-tyrosine, l-cysteic acid, cysteamine, l-cysteine sulfinic acid, n-acetyl-l-cysteine, s-methyl-l-cysteine, cystathionine, lanthionine, d,l-ethionine, n-acetyl-d,l- methionine, l-methionine sulfoxide, l-methionine sulfone, l-djenkolic acid, taurine, hypotaurine, phosphocreatine |
| Oligopeptides | 18 | l-alanyl-glycine, glycyl-l-aspartic acid, glycyl-l-glutamic acid, Ala-Asp, Ala-Gln, Ala-Glu, Ala-Gly, Ala-His, Ala-Leu, Ala-Thr, Gly-Asn, Gly-Gln, Gly-Glu, Gly-Met, Met-Ala, l-cysteinyl-glycine, glycyl-l-methionine, glutathione |
| Amines | 14 | phenylethyl-amine, putrescine, 2-aminoethanol, d,l-octopamine, methylamine, n-amylamine, n-butylamine, ethylamine, ethanolamine, ethylenediamine, agmatine, histamine, tyramine, sec-butylamine |
| Glycosides | 8 | β-methyl-d-glucoside, amygdalin, arbutin, α-methyl-d-glucoside, β-methyl-d-galactoside, α-methyl-d-mannoside, β-methyl-d-xyloside, salicin |
| Carboxylic acids | 19 | acetic acid, cis-aconitic acid, citric acid, formic acid, malonic acid, propionic acid, itaconic acid, sebacic acid, succinic acid, bromosuccinic acid, succinamic acid, butyric acid, capric acid, caproic acid, citraconic acid, citramalic acid, oxalic acid, oxalomalic acid, sorbic acid |
| Sugar acids | 10 | d-galactonic acid lactone, d-galacturonic acid, d-gluconic acid, d-glucosaminic acid, d-glucuronic acid, d-saccharic acid, n-acetyl-neuraminic acid, β-methyl-d-glucuronic acid, 5-keto-d-gluconic acid, melibionic acid |
| Modified carboxylic acids (hydroxy-, keto-, amino-) and esters | 20 | α-hydroxybutyric acid, β-hydroxybutyric acid, γ-hydroxybutyric acid, p-hydroxy phenylacetic acid, α-keto butyric acid, α-keto glutaric acid, α-keto valeric acid, d,l-lactic acid, 2-hydroxy benzoic acid, 4-hydroxy benzoic acid, d-tartaric acid, l-tartaric acid, d,l-α-amino-n-butyric acid, ε-amino-n-caproic acid, d,l-α-amino-caprylic acid, δ-amino-n- valeric acid, α-amino-n-valeric acid, pyruvic acid methyl ester, succinic acid mono-methyl-ester, d-lactic acid methyl ester |
| Nitrogen bases | 7 | adenine, cytosine, guanine, thymine, uracil, xanthine, alloxan |
| Nucleosides and nucleotides | 30 | inosine, uridine, thymidine, adenosine, cytidine, guanosine, xanthosine, adenosine-2'-monophosphate, adenosine-3'-monophosphate, adenosine-5'-monophosphate, adenosine-2',3'-cyclic monophosphate, adenosine-3',5'-cyclic monophosphate, guanosine-2'-monophosphate, guanosine-3'-monophosphate, guanosine-5'-monophosphate, guanisine-2',3'-cyclic monophosphate, guanisine-3',5'-cyclic monophosphate, cytidine-2'-monophosphate, cytidine-3'-monophosphate, cytidine-5'-monophosphate, cytidine-2',3'-cyclic monophosphate, cytidine-3',5'-cyclic monophosphate, uridine-2'-monophosphate, uridine-3'-monophosphate, uridine-5'-monophosphate, uridine-2',3'-cyclic monophosphate, uridine-3',5'-cyclic monophosphate, thymidine-3'-monophosphate, thymidine-5'-monophosphate, thymidine-3',5'-cyclic monophosphate |
| Inorganic compounds | 14 | ammonia, nitrite, nitrate, hydroxylamine, phosphate, pyrophosphate, trimetaphosphate, tripolyphosphate, hypophosphite, thiophosphate, dithiophosphate, sulfate, thiosulfate, tetrathionate, |
| Other organic compounds | 20 | urea, biuret, uric acid, thiourea, allantoin, parabanic acid, phosphoryl choline, o-phosphoryl-ethanolamine, phosphono acetic acid, 2-aminoethyl phosphonic acid, methylene diphosphonic acid, d,l-lipoamide, taurocholic acid, tetramethylene sulfone, quinic acid, d-ribono-1,4-lactone, p-amino benzene sulfonic acid, butane sulfonic acid, 2-hydroxyethane sulfonic acid, methane sulfonic acid |
