# Supplementary material for: Phenotype profiling of Rhizobium leguminosarum bv. trifolii clover nodule isolates reveal their both versatile and specialized metabolic capabilities
Source: Arch Microbiol. 2013 Feb 16;195(4):255–67. doi: 10.1007/s00203-013-0874-x (PMC3597991; doi:10.1007/s00203-013-0874-x)
Supplement: Supplementary file 3 — Supplementary material 3 (DOCX 25 kb) [file 203_2013_874_MOESM3_ESM.docx]

**Supplementary Table S3** PCA analyses: the names of variables, correlation coefficients between PCA factors and variables, and total variance in:

A) General PCA^G^ analysis

| **Groups of substrates** | **Factor loadings** | | | | | | |
| --- | --- | --- | --- | --- | --- | --- | --- |
|  | **PC1^G^** | **PC2^G^** | **PC3^G^** | **PC4^G^** | **PC5^G^** | **PC6^G^** | **PC7^G^** |
| **Monosaccharides** | **0.83** | 0.03 | -0.19 | 0.30 | -0.05 | 0.10 | 0.02 |
| **Oligosaccharides** | **0.87** | -0.26 | -0.13 | -0.09 | -0.05 | 0.11 | -0.15 |
| **Polysaccharides** | 0.19 | -0.05 | -0.58 | 0.00 | 0.69 | -0.09 | 0.09 |
| **Sugar alcohols** | **0.77** | 0.20 | 0.16 | 0.39 | -0.20 | -0.03 | 0.21 |
| **Modified sugars** | 0.08 | **0.70** | -0.03 | -0.05 | -0.05 | 0.40 | 0.33 |
| **Phospho-sugars** | -0.33 | -0.03 | 0.13 | -0.34 | 0.27 | 0.53 | 0.18 |
| **D-amino acids** | -0.04 | **0.81** | 0.18 | 0.01 | -0.07 | -0.32 | 0.05 |
| **L-amino acids** | 0.67 | 0.17 | 0.27 | -0.14 | 0.48 | 0.14 | 0.00 |
| **Modified amino acids** | -0.05 | 0.48 | 0.36 | -0.11 | 0.47 | -0.01 | 0.10 |
| **Oligopeptides** | -0.07 | 0.07 | 0.16 | -0.15 | -0.05 | 0.11 | **0.95** |
| **Amines** | 0.14 | **0.86** | 0.00 | -0.05 | 0.18 | 0.24 | -0.16 |
| **Glycosides** | 0.32 | 0.10 | 0.02 | 0.07 | -0.23 | **0.84** | 0.06 |
| **Carboxylic acids** | **0.66** | 0.37 | -0.33 | -0.05 | 0.00 | -0.10 | -0.34 |
| **Sugar acids** | 0.21 | -0.12 | 0.10 | **0.86** | -0.06 | 0.11 | -0.15 |
| **Modified carboxylic acids** | -0.32 | 0.28 | -0.36 | **0.73** | 0.12 | -0.12 | 0.14 |
| **Nitrogen bases** | -0.30 | 0.34 | 0.24 | **-0.68** | -0.07 | 0.20 | 0.21 |
| **Nucleosides and nucleotides** | -0.13 | 0.03 | 0.14 | 0.06 | **0.70** | -0.05 | -0.11 |
| **Inorganic compounds** | -0.10 | 0.21 | **0.78** | -0.02 | 0.15 | 0.38 | 0.02 |
| **Other organic compounds** | -0.04 | 0.02 | **0.83** | -0.16 | 0.09 | -0.21 | 0.24 |
| **Variance (%) of individual PCA^G^ component** | | | | | | | |
|  | 22.02 | 17.97 | 11.07 | 9.31 | 8.26 | 6.86 | 5.63 |

The remaining principal components resulted in 18,88% of metabolic variance of *Rlt* isolates

B) PCA^C^- PCA analysis of carbon sources utilization

| **Groups of substrates** | **Factor loadings** | | | | | |
| --- | --- | --- | --- | --- | --- | --- |
|  | **PC1^C^** | **PC2^C^** | **PC3^C^** | **PC4^C^** | **PC5^C^** | **PC6^C^** |
| **Monosaccharides** | **0,803** | 0,202 | 0,281 | 0,069 | 0,173 | 0,223 |
| **Oligosaccharides** | **0,713** | 0,368 | -0,046 | 0,032 | 0,469 | 0,059 |
| **Polysaccharides** | 0,203 | 0,216 | 0,031 | **-0,873** | 0,046 | -0,140 |
| **Sugar alcohols** | **0,646** | 0,334 | 0,329 | 0,326 | -0,141 | 0,161 |
| **Modified sugars** | 0,579 | 0,204 | -0,205 | 0,481 | -0,270 | 0,116 |
| **D-amino acids** | 0,308 | 0,258 | 0,380 | 0,458 | -0,017 | -0,504 |
| **L-amino acids** | 0,285 | **0,848** | 0,126 | -0,012 | 0,001 | 0,167 |
| **Modified amino acids** | 0,126 | 0,142 | 0,074 | 0,096 | **-0,895** | -0,091 |
| **Oligopeptides** | 0,077 | **0,744** | 0,004 | -0,247 | 0,011 | -0,547 |
| **Glycosides** | 0,192 | 0,109 | 0,060 | 0,140 | 0,103 | **0,904** |
| **Carboxylic acids** | **0,780** | 0,185 | -0,035 | -0,155 | -0,182 | -0,002 |
| **Sugar acids** | 0,152 | 0,213 | **0,839** | 0,152 | 0,125 | 0,027 |
| **Modified carboxylic acids** | -0,048 | -0,168 | **0,820** | -0,307 | -0,338 | -0,024 |
| **Nucleotides** | **0,868** | 0,121 | 0,061 | -0,143 | -0,105 | -0,113 |
| **Other organic compounds** | 0,339 | 0,835 | 0,015 | 0,013 | -0,159 | 0,018 |
| **Variance (%) of individual PCA^C^ component** | | | | | | |
|  | 34.6 | 13.4 | 11.5 | 11.5 | 7.7 | 6.6 |

C) PCA^N^- PCA analysis of nitrogen sources utilization

| **Groups of substrates** | **Factor loadings** | | | | |
| --- | --- | --- | --- | --- | --- |
|  | **PC1^N^** | **PC2^N^** | **PC3^N^** | **PC4^N^** | **PC5^N^** |
| **Modified sugars** | **0,855** | 0,014 | -0,211 | 0,146 | -0,017 |
| **D-amino acids** | 0,384 | -0,146 | -0,019 | **0,680** | 0,338 |
| **L-amino acids** | **0,738** | 0,219 | 0,133 | 0,215 | 0,137 |
| **Modified amino acids** | 0,107 | 0,122 | 0,046 | **0,907** | -0,065 |
| **Oligopeptides** | 0,093 | -0,022 | **-0,919** | -0,036 | 0,141 |
| **Amines** | **0,723** | -0,235 | 0,418 | 0,402 | -0,146 |
| **Modified carboxylic acids** | 0,068 | 0,011 | -0,130 | 0,065 | **0,959** |
| **Nitrogen bases** | **0,655** | 0,549 | -0,223 | -0,060 | 0,223 |
| **Nucleosides** | 0,247 | **0,821** | 0,093 | 0,105 | -0,258 |
| **Inorganic compounds** | 0,388 | **0,648** | 0,454 | -0,048 | 0,088 |
| **Other organic compounds** | -0,204 | **0,907** | -0,118 | -0,022 | 0,135 |
| **Variance (%) of individual PCA^N^ component** | | | | | |
|  | 31.6 | 19.9 | 15.2 | 8.9 | 7.6 |

D) PCA^P/S^- PCA analysis of phosphorous and sulfur sources utilization

| **Groups of substrates** | **Factor loadings** | | | |
| --- | --- | --- | --- | --- |
|  | **PC1^P/S^** | **PC2^P/S^** | **PC3^P/S^** | **PC4^P/S^** |
| **Phospho-sugars** | **0,675** | -0,566 | 0,107 | -0,217 |
| **D-amino acids** | 0,041 | 0,046 | **0,898** | 0,342 |
| **L-amino acids** | 0,316 | **0,777** | 0,230 | -0,069 |
| **Modified amino acids** | 0,239 | **-0,790** | 0,147 | 0,287 |
| **Oligopeptides** | 0,159 | -0,055 | **0,816** | -0,347 |
| **Nucleotides** | 0,177 | -0,205 | 0,041 | **0,822** |
| **Inorganic compounds** | **0,827** | 0,049 | 0,052 | 0,226 |
| **Other organic compounds** | **0,645** | 0,141 | 0,557 | 0,221 |
| **Variance (%) of individual PCA^P/S^ component** | | | | |
|  | 33.7 | 21.8 | 12.6 | 11.9 |
